# Supplementary figures and images for: A Foundational Population Genetics Investigation of the Sexual Systems of Solanum (Solanaceae) in the Australian Monsoon Tropics Suggests Dioecious Taxa May Benefit from Increased Genetic Admixture via Obligate Outcrossing
Source: Plants (Basel). 2023 Jun 2;12(11):2200. doi: 10.3390/plants12112200 (PMC10255629; doi:10.3390/plants12112200)

**asym – Isolation by distance plot**

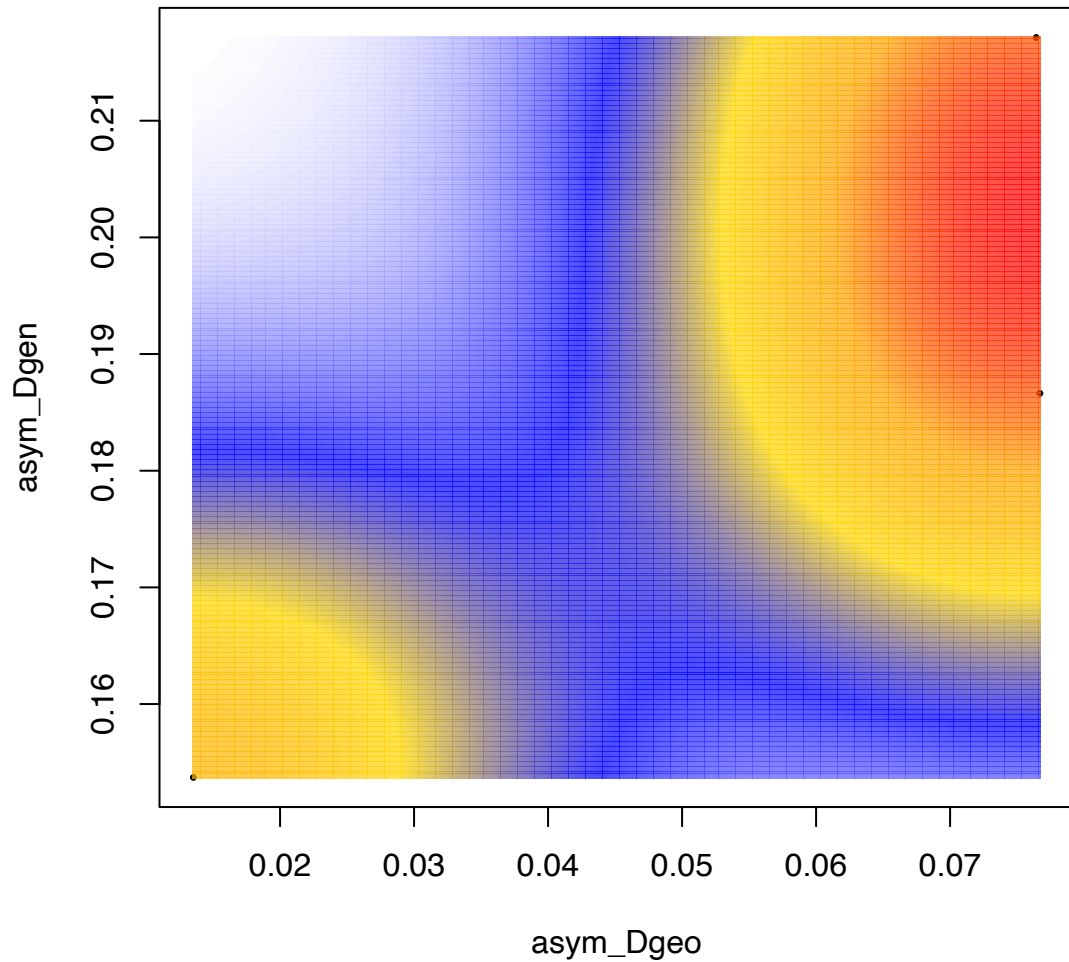

**raphi – Isolation by distance plot**

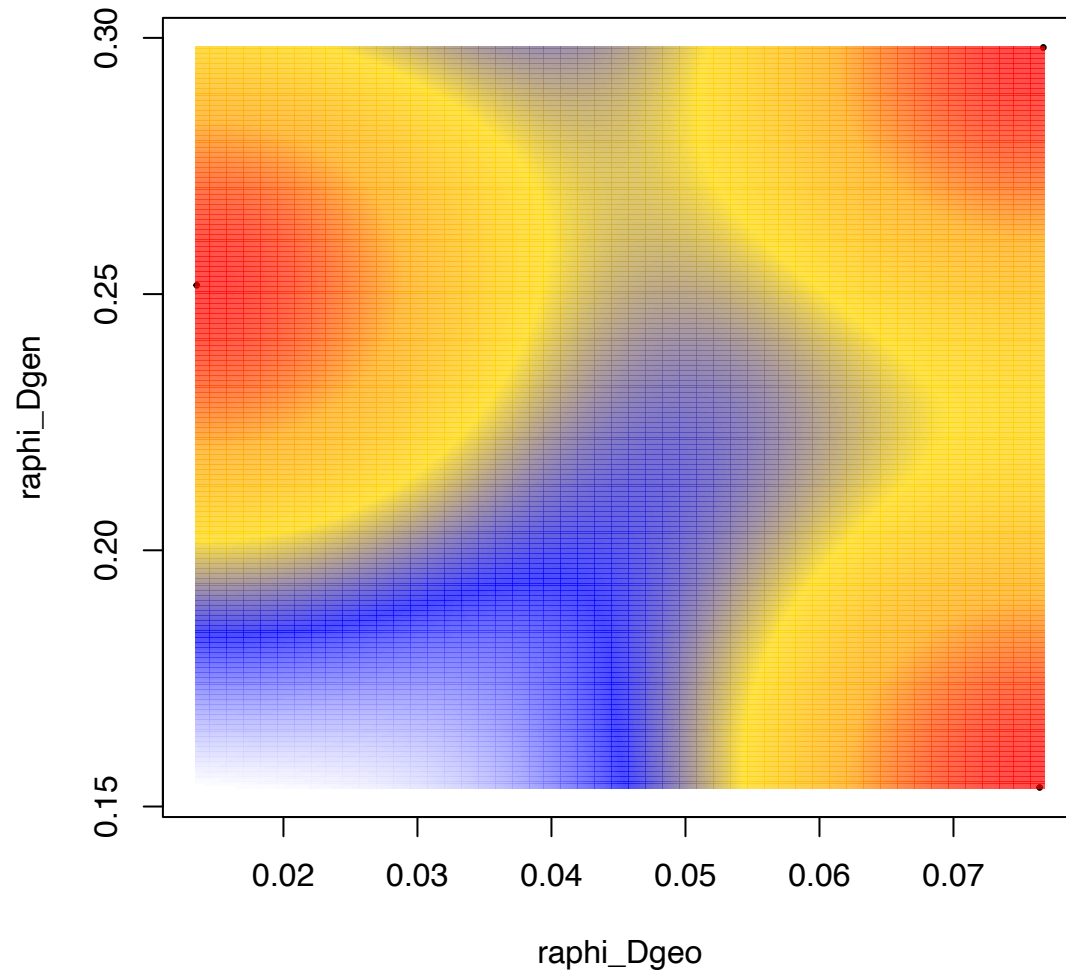

Supplement: Supplementary file 1 [file plants-12-02200-s001.zip › plants-2270133-supplementary.pdf]
